# Reduced incidence of ventilator-associated pneumonia (VAP) at a tertiary care center due to enhanced compliance with evidence-based bundle care practices through structured training programs for ICU healthcare workers: a pilot study

**DOI:** 10.3205/dgkh000669

**Published:** 2026-07-28

**Authors:** Dasari Spurthy, Chetana Guddadahalli Shankrappa, Syeda Saba Hashmiya, Manisha Mane, Prathyusha Pedapati

**Affiliations:** 1 Department of Microbiology, ESIC Medical College and Hospital, Hyderabad, Telangana, India

**Keywords:** ventilator-associated pneumonia, VAP bundle, ICU infection control, hospital-acquired infections

## Abstract

**Background:**

Ventilator-associated pneumonia (VAP) is among the most prevalent hospital-acquired infections in ICU settings, significantly contributing to increased morbidity, mortality, and healthcare costs. This study aimed to reduce the incidence of VAP by enhancing compliance with evidence-based bundle care practices through structured training programs for ICU healthcare workers.

**Methods:**

A quasi-experimental, prospective study was conducted in Medical and Respiratory ICUs over six months (October 2024 – March 2025). Phase 1 involved baseline assessment through direct observations and questionnaires, followed by intensive Hospital Infection Prevention and Control (HIPC) training sessions. Phase 2 reinforced the implementation of bundle care protocols with periodic audits and feedback. Data was collected on compliance rates and incidence of VAP.

**Results:**

VAP incidence was expressed as standardized rates per 1,000 ventilator days. Rates decreased from 10, 16.4 and 16 per 1,000 ventilator-days during the pre-intervention phase, October–December 2024 to 7.4, 5.3, and 4.9 per 1,000 ventilator-days, respectively, during the post-intervention phase January–March 2025, demonstrating sustained improvement following bundle compliance interventions. Survey scores post-intervention averaged 68%, indicating improved knowledge and adherence.

**Conclusion:**

Structured training and consistent reinforcement of VAP bundle practices were associated with improved compliance and a sustained downward trend in VAP incidence. This approach underscores the importance of targeted education and real-time audits in improving infection control practices.

## Zusammenfassung

**Hintergrund:**

Die beatmungsassoziierte Pneumonie (VAP) zählt zu den häufigsten nosokomialen Infektionen auf Intensivstationen und trägt erheblich zu erhöhter Morbidität, Mortalität und höheren Gesundheitskosten bei. Ziel der Studie war es, die Inzidenz der VAP zu senken, indem die Einhaltung eines evidenzbasierten Maßnahmebündels durch strukturierte Schulungsprogramme für das Pflegepersonal verbessert wurde.

**Methode:**

Über einen Zeitraum von sechs Monaten (Oktober 2024 – März 2025) wurde eine quasi-experimentelle, prospektive Studie auf medizinischen und pulmonologischen Intensivstationen durchgeführt. In Phase 1 erfolgte eine Baseline-Erhebung mittels direkter Beobachtungen und Fragebögen, gefolgt von intensiven Schulungen zur Prävention und Bekämpfung von Krankenhausinfektionen. In Phase 2 wurde die Umsetzung der Bundle-Care-Protokolle durch regelmäßige Audits und Feedback verstärkt. Es wurden Daten zu den Einhaltungsquoten und zur Inzidenz von VAP erhoben.

**Ergebnisse:**

Die VAP-Inzidenz wurde als standardisierte Rate pro 1.000 Beatmungstage angegeben. Die Raten sanken von 10, 16,4 bzw. 16 pro 1.000 Beatmungstage in der Phase vor der Intervention (Oktober–Dezember 2024) auf 7,4, 5,3 bzw. 4,9 pro 1.000 Beatmungstage in der Phase nach der Intervention (Januar–März 2025), was eine anhaltende Verbesserung nach den Maßnahmen zur Einhaltung des Maßnahmenpakets zeigt. Die Umfrageergebnisse nach der Intervention lagen im Durchschnitt bei 68 %, was auf ein verbessertes Wissen und eine verbesserte Einhaltung hinweist.

**Schlussfolgerung:**

Ein strukturiertes Schulungsprogramm und die konsequente Umsetzung der Maßnahmen zur Prävention von VAP gingen mit einer verbesserten Einhaltung der Vorschriften und einem anhaltenden Rückgang der VAP-Inzidenz einher. Dieser Ansatz unterstreicht die Bedeutung gezielter Schulungen und Echtzeit-Audits für die Verbesserung der Infektionskontrollmaßnahmen.

## Introduction

Ventilator-associated pneumonia (VAP) is one of the most common and serious hospital-acquired infections affecting critically-ill patients receiving invasive mechanical ventilation in intensive care units (ICUs). It typically develops after 48 hours of endotracheal intubation and is associated with significant morbidity, mortality, prolonged mechanical ventilation, increased ICU and hospital length of stay, and higher healthcare costs [1], [2], [3]. Owing to these adverse outcomes, prevention of VAP has become a major patient-safety priority and an important quality-of-care indicator in critical care settings [1], [4].

The epidemiology and burden of VAP vary widely across regions, patient populations, and ICU settings. Multicenter studies have reported VAP incidence ranging from 3.5 to over 40 episodes per 1,000 ventilator days, with higher rates consistently observed in low- and middle-income countries [2], [5]. Large observational cohorts have demonstrated that VAP and ventilator-associated events are linked to significantly longer ICU and hospital stays and more than a three-fold increase in mortality compared with non-affected ventilated patients [3]. Prolonged mechanical ventilation, repeated intubation, tracheostomy, hypoproteinemia, prior antibiotic exposure, and advanced age have been identified as important risk factors for VAP development [3], [6].

The pathogenesis of VAP is multifactorial and closely related to the disruption of normal host defense mechanisms caused by endotracheal intubation. Micro-aspiration of colonized oropharyngeal secretions, impaired mucociliary clearance, biofilm formation on endotracheal tubes, and extended duration of ventilation contribute significantly to infection development [2], [3]. Common causative organisms include *Staphylococcus aureus*, *Pseudomonas aeruginosa*, and *Klebsiella* *pneumoniae*, with late-onset VAP frequently associated with multidrug-resistant pathogens, complicating treatment and worsening outcomes [2], [6]. 

Given the preventable nature of many VAP risk factors, evidence-based ventilator care bundles have been widely promoted as a cornerstone of VAP prevention strategies. These bundles typically include head-of-bed elevation, daily sedation interruption with assessment of readiness to extubate, oral care with antiseptics, subglottic secretion drainage, endotracheal pressure monitoring, and strict hand hygiene [1], [7], [8]. Multiple studies have demonstrated that consistent implementation of ventilator bundles significantly reduces VAP incidence, ventilator days, ICU length of stay, and overall healthcare burden [1], [7].

Despite strong evidence supporting bundle-based prevention, real-world compliance remains suboptimal in many ICUs. Audits and observational studies have identified gaps in staff knowledge, negative attitudes toward bundle implementation, inconsistent education, and inadequate documentation as major barriers to effective VAP prevention [8], [9], [10]. Studies from tertiary care centers in India have highlighted that although awareness of individual bundle components is often high, adherence to all components simultaneously and proper documentation remain inconsistent, limiting the overall effectiveness of prevention strategies [8], [9].

Quality-improvement initiatives focusing on structured education, standardized checklists, continuous auditing, and documentation reinforcement have shown promising results. Implementation of Plan-Do-Study-Act (PDSA) cycles, simulation-based training, and documentation standardization have been associated with sustained improvements in bundle compliance and significant reductions in VAP rates [1], [7]. These findings underscore the importance of continuous monitoring, education, and system-level interventions to achieve durable improvements in patient outcomes. In this context, the present study undertakes a scrupulous evaluation of VAP among ICU patients in a tertiary care center, with a focus on enhancing VAP bundle compliance, staff education, and documentation practices. By identifying gaps in current practices and implementing targeted quality improvement measures, this study aims to contribute to improved infection control, patient safety, and clinical outcomes in critically-ill mechanically ventilated patients. Despite widespread adoption of ventilator care bundles, most published studies focus primarily on either bundle implementation or outcome reduction, with limited emphasis on integrated evaluation of compliance, education, documentation, and standardized surveillance metrics within routine ICU practice. Additionally, data from resource-limited tertiary care settings in India, using ventilator-days-based incidence reporting, remain scarce.

The novelty of the present study lies in its structured, phased quality improvement approach that simultaneously targets staff education, real-time compliance auditing, documentation reinforcement, and conversion of VAP reporting from percentages to standardized rates per 1,000 ventilator days. This integrated framework provides a pragmatic and reproducible model for infection prevention in similar ICU settings.

## Materials and methods

### Study design and setting

This was designed as a prospective study conducted over six months from October 2024 to March 2025 at the Medical ICU (MICU) and Respiratory ICU (RICU) at ESIC Medical College & Hospitals, Hyderabad. The study protocol was approved by the Institutional Ethics Committee (Approval No.: ESICMC/SNR/IEC-F674/02-2025). As this was an observational study using routinely collected clinical data, patient confidentiality was maintained throughout the study.

### Study population

The study population consisted of adult patients (18–60 years) admitted to MICU and RICU, requiring mechanical ventilation for more than 48 hours. Patients below 18 years and elderly patients above 60 years were excluded to reduce confounding due to age-related comorbidities and differing physiological responses that may independently influence the risk of VAP. Patients on continuous positive airway pressure, nasal prongs, or transferred with ventilators in-situ from other hospitals were excluded.

### Sample size

The VAP rate was calculated using the number of ventilator days as the denominator. Ventilator days were defined as the total number of days that patients were on mechanical ventilation during the study period.

To ensure adequate representation, all patients who were on mechanical ventilation for more than 48 hours during the study period were included in the study, resulting in a total of 40 patients. The criteria used to diagnose VAP were not explicitly standardized across surveillance periods, and VAP incidence was reported primarily as percentages rather than standardized rates (e.g., per 1,000 ventilator days), thereby limiting direct comparability with existing studies. VAP incidence was recalculated and expressed as rates per 1,000 ventilator days using the standard formula: 


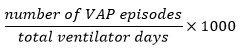


This standardized reporting allows direct comparison with national and international studies and aligns with established surveillance recommendations.

VAP was diagnosed based on clinical, radiological, and microbiological criteria in accordance with standard guidelines. Clinical criteria included fever, leukocytosis, purulent tracheal secretions, and worsening oxygenation in patients receiving mechanical ventilation for more than 48 hours. Radiological diagnosis was supported by new or progressive infiltrates on chest radiography. Microbiological confirmation was obtained through endotracheal aspirate culture demonstrating significant bacterial growth in accordance with criteria suggested by the Centers for Disease Control and Prevention (CDC).

### Study phase 1 (October–December 2024)

Baseline compliance was assessed through structured questionnaires (Table S1 in Attachment 1) and direct observation. Twenty (20) ICU nurses completed the initial survey, achieving an average score of 68%.

Training sessions were conducted for ICU nurses and staff focusing on the VAP bundle. The sessions included interactive lectures, bedside demonstrations, and practical skill training covering hand hygiene, head-of-bed elevation (30 degrees), oral hygiene with antiseptic solution, subglottic suctioning, sedation holiday, and daily assessment for extubating readiness. Each session lasted 45–60 minutes, and multiple sessions were conducted over a two-week period to ensure participation of all ICU staff.

A three-month baseline period was selected to establish pre-intervention compliance levels and VAP incidence trends, allowing adequate observation time to capture routine ICU practices before implementation of reinforcement measures.

### Study phase 2 (January–March 2025)

This included the following activities:

- Reinforcement of bundle practices with regular audits.
- Immediate feedback interventions to address compliance lapses.
- Continuous data collection on adherence and VAP incidence.

Unlike prior studies focusing on isolated bundle components, this study employed a multidimensional intervention combining education, bedside audits, documentation review, and standardized outcome measurement, enabling simultaneous assessment of process and outcome indicators.

Nursing documentation of VAP-bundle components improved during Phase 2 as a result of regular monitoring and feedback provided during bedside audits (Table 1 (Tab. 1)).

### Data collection tools

Compliance with VAP-bundle practices was assessed through daily bedside audits using a standardized checklist, direct observation by infection control personnel, and review of nursing documentation. Compliance was calculated as the percentage of correctly performed bundle components out of the total observed opportunities. Although 20 nurses participated in the training program, multiple bedside observations were conducted during routine audits to evaluate adherence to bundle practices. Therefore, 40 total observations of nursing practices were recorded during the evaluation of bedside performance.

### Statistical analysis

Data was analyzed using SPSS version 22. Descriptive statistics were used to summarize compliance rates and VAP incidence. Pre- and post-intervention VAP rates (per 1,000 ventilator days) were compared using appropriate inferential statistical tests. A rate comparison test (Poisson test/chi-squared test for incidence rates) was applied to assess differences between phases. 95% confidence intervals (CI) were calculated for VAP rates. A p-value <0.05 was considered statistically significant.

## Results

The baseline characteristics of the study population were analyzed. Among the 40 patients, the mean age was 40 ± 5 years, with 27 males and 13 females. The most common indications for mechanical ventilation were respiratory failure, sepsis, and neurological impairment. The mean duration of mechanical ventilation was 15–20 days, and the average ICU stay was 30 days.

### Impact of training

A total of 20 ICU nurses participated in the training program during Phase 1. Baseline assessment using a structured questionnaire showed an average knowledge score of 68% regarding VAP-bundle practices. Following the training sessions and reinforcement audits in Phase 2, knowledge and adherence to bundle components improved significantly. Compliance with key bundle elements. such as hand hygiene, head-of-bed elevation, cuff pressure monitoring, and documentation of care, showed marked improvement during the post-intervention period.

Each component of oral care was evaluated individually during bedside audits, including cleaning of the endotracheal tube, mucous membranes, palate, and teeth. Therefore, observations for these components were recorded separately in the evaluation (Table 2 (Tab. 2)).

Comparison of baseline and post-intervention data demonstrated improved compliance with VAP prevention practices following the educational intervention. Hand hygiene compliance increased from baseline questionnaire knowledge levels of 68% to observed compliance of 97.5%, while adherence to head-of-bed elevation improved to 97.5%. Oral care practices also improved substantially, although initial remediation was required for certain components such as endotracheal tube cleaning. Documentation of ventilator bundle practices also improved following the reinforcement sessions and regular audits.

**Figure 1:**
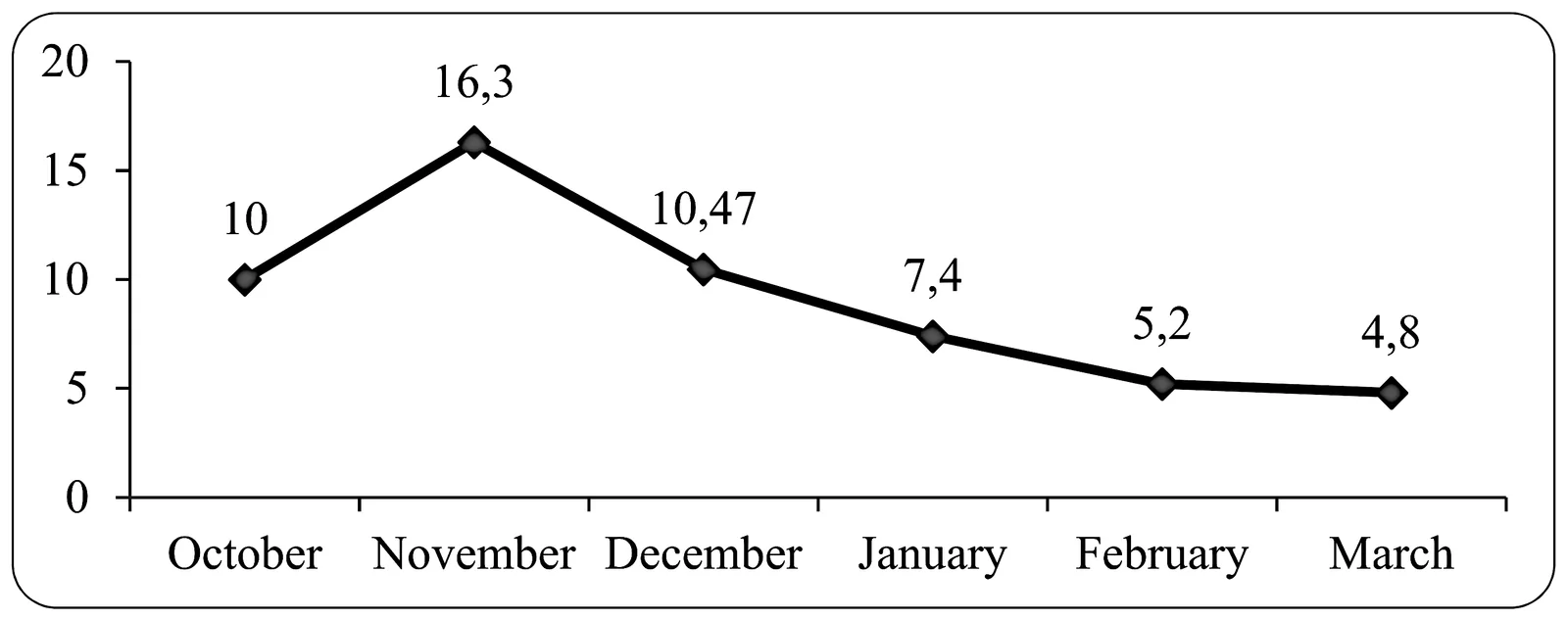
VAP Trend (Oct 2024 – Mar 2025)

Following Phase 1 and Phase 2 interventions, compliance steadily improved, and VAP incidence showed a progressive decline from 10 per 1,000 ventilator days in October to 4.8 per 1,000 ventilator days in March, reflecting improved compliance with ventilator bundle practices following training and reinforcement interventions (Figure 1 (Fig. 1)).

The monthly VAP incidence demonstrated a gradual reduction following implementation of the educational intervention and compliance monitoring, suggesting improved adherence to ventilator bundle practices over the study period.

The key observations were:

- Improved adherence to hygiene protocols and documentation,
- enhanced knowledge and awareness among nursing staff and
- a sustained reduction in VAP incidence following implementation of the intervention.

## Discussion

In the present study, the observed burden of VAP and the improvement seen following targeted interventions reinforce existing evidence from earlier studies that systematic implementation of VAP bundles is associated with reductions in VAP incidence [1], [6], [10], [11], findings that are consistent with the trends observed in the present study. 

Our findings support those of earlier studies demonstrating that standardized, evidence-based VAP prevention protocols lead to meaningful reductions in VAP rates. Buterakos et al. [1] reported a significant decline in VAP following implementation of a unit-wide bundle and structured nursing education, emphasizing the critical role of compliance and documentation in sustaining improvements. Similar reductions have been observed in multicenter and single-center quality improvement initiatives, where improvements in bundle adherence translated into reduced ventilator days and ICU length of stay [1], [10], [11].

The burden of VAP observed in our ICU aligns with international and regional epidemiological data. Multicenter studies have consistently demonstrated that VAP is associated with prolonged mechanical ventilation, extended ICU and hospital stays, and increased mortality [3], [7], [9]. A Portuguese eVAP-PT study highlighted the substantial healthcare burden imposed by VAP, including increased antimicrobial use and resource consumption [7], findings that mirror trends observed in other large observational cohorts [3], [9].

One of the major challenges in VAP prevention remains the complexity of diagnosis and surveillance. Traditional VAP definitions are subjective and prone to interobserver variability, prompting the adoption of ventilator-associated events (VAE) as a more objective surveillance measure in some healthcare systems [3], [4]. Studies evaluating VAE surveillance have demonstrated strong associations with adverse outcomes and increased antibiotic utilization, supporting its role as a complementary quality indicator [4]. However, VAP-focused prevention bundles remain clinically relevant, particularly in settings where bundle compliance directly influences bedside care practices [1], [6].

Risk factor analysis from prior studies supports the emphasis placed on modifiable preventive strategies in the present study. Prolonged mechanical ventilation, repeated intubation, tracheostomy, prior antibiotic exposure, and poor nutritional status have been repeatedly identified as independent predictors of VAP [5], [8]. Nomogram-based risk prediction models further reinforce the importance of early identification of high-risk patients and targeted preventive interventions [8], underscoring the value of structured ICU protocols.

Despite strong evidence supporting VAP bundles, compliance remains inconsistent across ICUs. Audits and observational studies from both developed and developing countries demonstrate that adherence to all bundle components simultaneously is often suboptimal [12], [13], [14]. Studies from Indian tertiary care centers have shown that while individual components such as head-of-bed elevation are widely practiced, other elements, e.g., daily sedation interruption, subglottic suctioning, and documentation, are frequently overlooked [10], [12], [13], [14]. These findings parallel the gaps identified in our baseline assessments. Education and staff engagement emerged as key drivers of improved compliance in our study, consistent with published literature. Multiple studies have demonstrated that educational interventions alone are insufficient unless reinforced through audits, feedback, standardized checklists, and documentation tools [6], [10], [12], [11]. Quality improvement initiatives using Plan–Do–Study–Act cycles and multidisciplinary involvement have shown sustained improvements in bundle adherence and VAP reduction [10], [11], supporting the multifaceted approach adopted in our setting.

Documentation plays a pivotal role in ensuring accountability and continuity of care. Studies auditing VAP-bundle practices have emphasized that incomplete or inconsistent documentation undermines both surveillance accuracy and quality improvement efforts [1], [6], [13]. Improved documentation not only facilitates monitoring of compliance but also supports antimicrobial stewardship by reducing unnecessary or prolonged antibiotic exposure [4], [15]. 

## Limitations

The findings of this study should be interpreted in light of certain limitations. The number of patients (40) and nurses included (20) in this study was very small. Given the quasi-experimental single-center design and absence of a parallel control group, the findings should be interpreted as associative rather than causal, and claims of long-term effectiveness should be viewed cautiously. Additionally, reliance on retrospective data and documentation accuracy may introduce information bias. However, similar methodological constraints are noted in many published VAP quality improvement studies [1], [6], [10], and the consistency of our findings with existing literature strengthens their validity.

Overall, this study adds to the growing body of evidence demonstrating that VAP is a preventable ICU complication when evidence-based bundles are strictly and correctly implemented. Sustained improvements require not only protocol availability but also continuous education, structured audits, and robust documentation practices. Future research should focus on long-term sustainability, integration of electronic surveillance tools, and assessment of patient-centered outcomes.

## Conclusion

In this single-center quality improvement study, implementation of structured HIPC training and bundle reinforcement was associated with improved compliance and a consistent decline in VAP incidence. These findings support the role of education, auditing, and documentation in strengthening infection prevention practices, while highlighting the need for larger controlled studies to confirm long-term effectiveness.

## Notes

### Authors’ ORCIDs

- Spurthy D: https://orcid.org/0009-0004-8037-2011
- Chetana GS: https://orcid.org/0000-0002-2639-2888
- Hashmiya SS: https://orcid.org/0009-0002-2725-034
- Mane M: https://orcid.org/0000-0001-7658-9497
- Pedapati P: https://orcid.org/0009-0005-2274-6190

### Ethical approval 

The study protocol was approved by the institutional ethics committee.

### Funding

None. 

### Acknowledgments

All authors express their gratitude to the ICU nursing staff, respiratory therapists and infection control team for their cooperation during data collection and implementation of the educational interventions. Special thanks to the Dean and the Institutional Ethics Committee for their support and guidance throughout the study.

### Competing interests

The authors declare that they have no competing interests.

## Supplementary Material

Table S1

## Figures and Tables

**Table 1:**
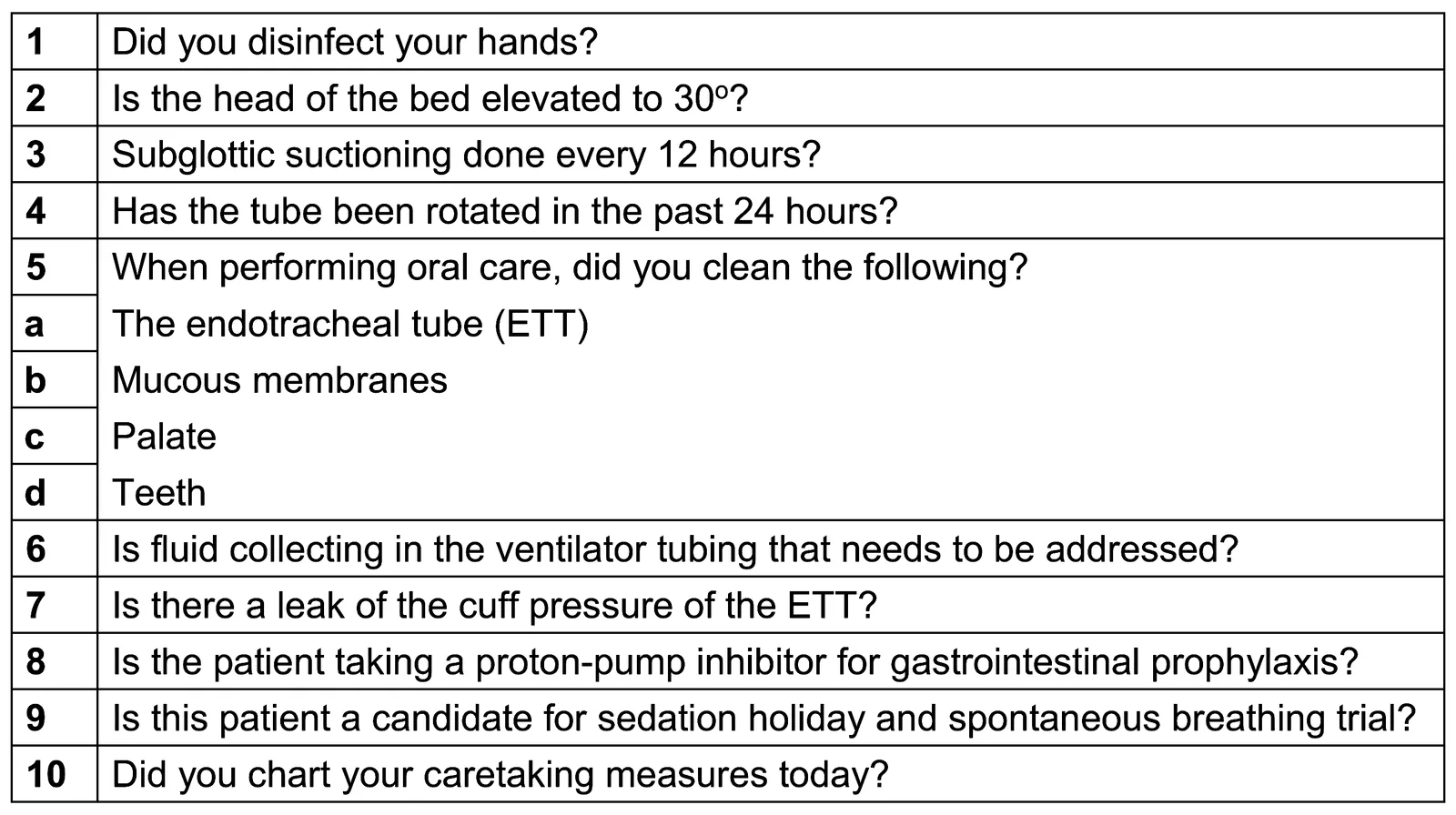
VAP prevention check list

**Table 2:**
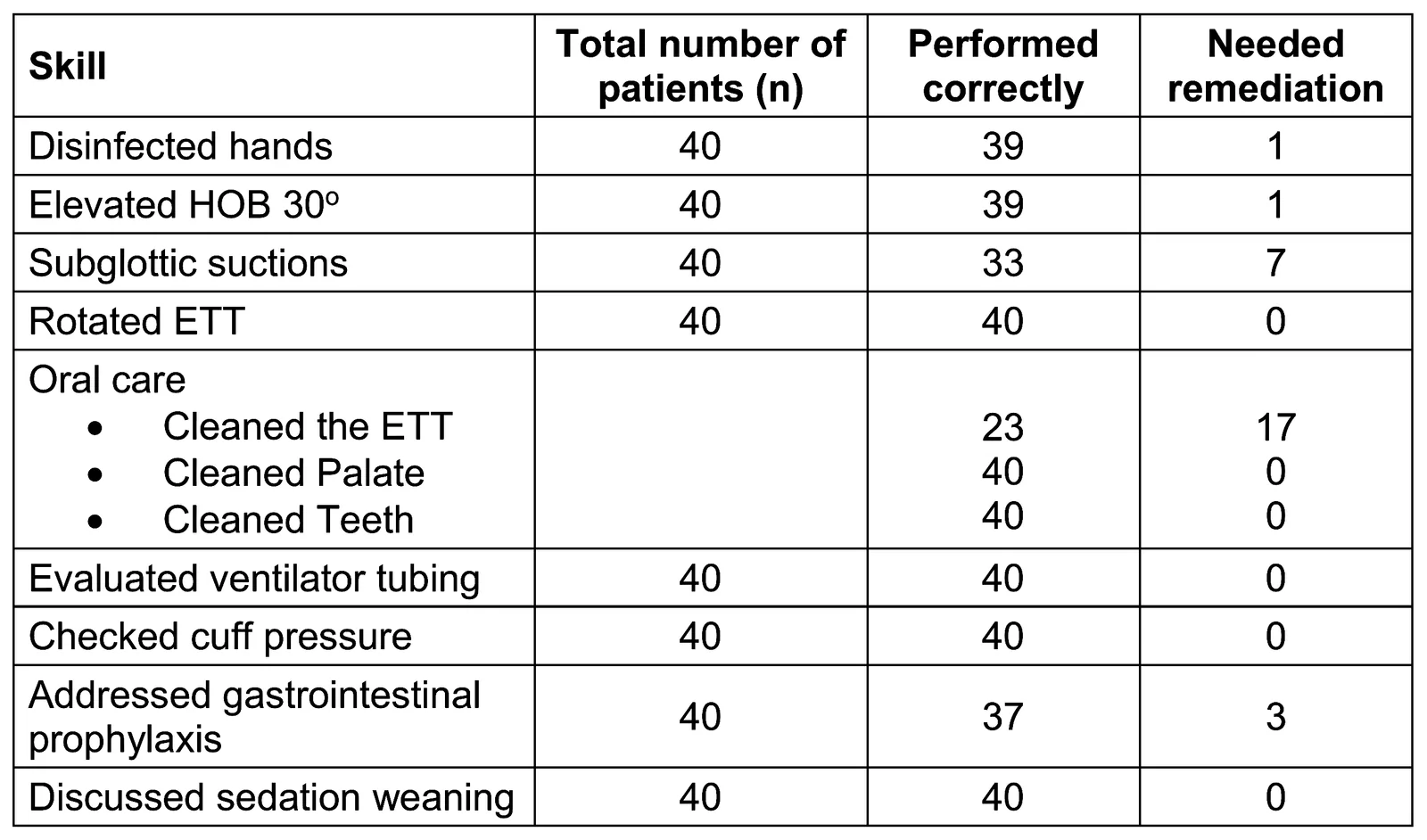
Evaluation of bedside performance following training
