# Supplementary material for: Reduced incidence of ventilator-associated pneumonia (VAP) at a tertiary care center due to enhanced compliance with evidence-based bundle care practices through structured training programs for ICU healthcare workers: a pilot study
Source: GMS Hyg Infect Control. 2026 Jul 28;21:Doc60. doi: 10.3205/dgkh000669 (PMC13585034; doi:10.3205/dgkh000669)
Supplement: Supplementary file 1 [file HIC-21-60-s-001.pdf]

Attachment 1

Table S1

1. Which of the following are clinical signs of VAP?<br>☐ Fever<br>☐ Hypothermia<br>☐ Bradycardia<br>☐ Purulent secretions	11. How does frequent suctioning relate to VAP?<br>a) Reduces the risk<br>b) Increases the risk if not sterile<br>c) Has no effect<br>d) Prevents all infections
2. What white blood cell count range is considered suggestive of infection in VAP?<br>a) <2,000/mm^3<br>b) <4,000 or >12,000/mm^3<br>c) 5,000–10,000/mm^3<br>d) >20,000/mm^3	12. True or false: Biofilm on the endotracheal tube can harbor pathogens and contribute to VAP.<br>☐ True<br>☐ False
3. A sudden drop in oxygen saturation and increased ventilator requirements may indicate:<br>a) Recovery<br>b) VAP<br>c) Hemorrhage<br>d) Dehydration	13. Reintubation increases the risk of VAP.<br>☐ True<br>☐ False
4. Purulent tracheal secretions are typically:<br>a) Clear and watery<br>b) Green/yellow and thick<br>c) Bloody<br>d) Odorless and clear	14. Prior use of antibiotics can:<br>a) Prevent VAP completely<br>b) Increase the risk of resistant organisms<br>c) Have no effect<br>d) Cure VAP before it starts
5. True or false: A new onset of cough or dyspnea can be a clinical sign of VAP.<br>☐ True ☐ False	15. Why is patient positioning important in VAP prevention?<br>a) Reduces aspiration<br>b) Helps sleep<br>c) Improves heart rate<br>d) Lowers blood pressure
6. Which of the following is a clinical condition where head-of-bed elevation may not be recommended as part of VAP prevention?<br>a) Mechanically ventilated patient with stable hemodynamics<br>b) Patient with spinal cord injury and cervical spine instability<br>c) post-operative abdominal surgery patient with no complications<br>d) Patient on prolonged sedation without contraindications	16. What is the recommended head-of-bed elevation to help prevent aspiration?<br>a) 10–20 degrees<br>b) 25–30 degrees<br>c) 30–45 degrees<br>d) 60–90 degrees

Attachment 1 to: *Spurthy D, Shankrappa CG, Hashmiya SS, Mane M, Pedapati P. Reduced incidence of ventilator-associated pneumonia (VAP) at a tertiary care center due to enhanced compliance with evidence-based bundle care practices through structured training programs for ICU healthcareworkers: a pilot study. GMS Hyg Infect Control. 2026;21:Doc60.*
DOI: 10.3205/dgkh000669, URN: urn:nbn:de:0183-dgkh0006699

7. What is a common diagnostic tool for detecting VAP pathogens?<br>a) Urine culture<br>b) Bronchoalveolar lavage (BAL)<br>c) Sputum test only<br>d) ECG	17. Name one method of reducing aspiration risk in ventilated patients:
8. Which pathogens are commonly associated with VAP?<br>☐ MRSA<br>☐ *Escherichia coli*<br>☐ *Pseudomonas aeruginosa*<br>☐ *Candida albicans*	18. List of two practices that can reduce the incidence of VAP in ventilated patients:
9. True or false: A quantitative culture from a protected specimen brush (PSB) is a valid method to diagnose VAP.<br>☐ True ☐ False	19. Why is head-of-bed elevation included in the VAP bundle?<br>a) To prevent aspiration of gastric contents<br>b) To reduce intracranial pressure<br>c) To enhance cardiac output<br>D. To improve oxygen diffusion
10. How many hours of mechanical ventilation does VAP risk significantly increase?<br>a) 12 hours<br>b) 24 hours<br>c) 48 hours<br>d) 72 hours	20. What role does oral care with chlorhexidine play in preventing VAP?<br>a) It reduces oral bacterial colonization that can lead to pneumonia<br>b) It prevents tooth decay in intubated patients<br>c) It improves patient satisfaction scores<br>d) It maintains moisture in the oral cavity

Attachment 1 to: *Spurthy D, Shankrappa CG, Hashmiya SS, Mane M, Pedapati P. Reduced incidence of ventilator-associated pneumonia (VAP) at a tertiary care center due to enhanced compliance with evidence-based bundle care practices through structured training programs for ICU healthcareworkers: a pilot study. GMS Hyg Infect Control. 2026;21:Doc60.*
DOI: 10.3205/dgkh000669, URN: urn:nbn:de:0183-dgkh0006699
